# Supplementary material for: Genetic Architecture and Genome-Wide Adaptive Signatures Underlying Stem Lenticel Traits in Populus tomentosa
Source: Int J Mol Sci. 2021 Aug 26;22(17):9249. doi: 10.3390/ijms22179249 (PMC8431110; doi:10.3390/ijms22179249)
Supplement: Supplementary file 1 [file ijms-22-09249-s001.zip › ijms-1313588-supplementary.pdf]

# Genetic architecture and genome-wide adaptive signatures underlie stem lenticel in *Populus tomentosa*

Peng Li[1,2,3#], Jiaxuan Zhou[1,2,3#], Dan Wang[1,2,3], Liangzheng Li[1,2,3], Liang Xiao[1,2,3], Mingyang Quan[1,2,3], Wenjie Lu, Liangcheng Yao, Yuanyaun Fang, Chenfei Lv, Fangyuan Song, Qingzhang Du[1,2,3], and Deqiang Zhang[1,2,3,*]

**The following Supporting Information is available for this article:**

**Supporting Figures:**

**Figure S1** Characterization of lenticel in *P. tomentosa*.

**Figure S2** Phenotypic variations in the lenticels of the *Populus tomentosa* population.

**Figure S3** The expression of *PtoNAC083* and *PtoMYB46* under drought and normal conditions in a population constituted with 100 accessions of *P. tomentosa*.

**Supporting Tables:**

**Table S1** Statistical analysis of three lenticel traits of *Populus tomentosa*.

**Table S2** Correlations of lenticel area (LA), lenticel number (LN), and the ratio of the total LA to the area of a standard rectangular sampling window (RA) with the diameter at breast height (DBH) of *Populus tomentosa*.

**Table S3** Analysis of variance of phenotypic variation in the three subpopulations.

**Table S4** Multiple comparisons of phenotype data from different subpopulations.

**Table S5** Climate factors in the three subgroups.

**Table S6** Full details of genome-wide association studies (GWAS) of *Populus tomentosa* lenticels, indicating single nucleotide (SNP) information, associated *P*

values, additive and dominant effects, marker $r^2$ values, and *Arabidopsis* homolog and putative gene functions.

**Table S7** Gene ontology (GO) analysis of 88 genes in genome-wide association studies (GWAS)

**Table S8** Detailed information on significant epistatic SNP pairs for lenticel traits from the GWAS results for *Populus tomentosa*.

**Table S9** Expression data for several genes detected by GWAS in different tissues of *Populus*.

**Table S10** Gene-specific primers used in this study.

**1 Supporting Figures:**

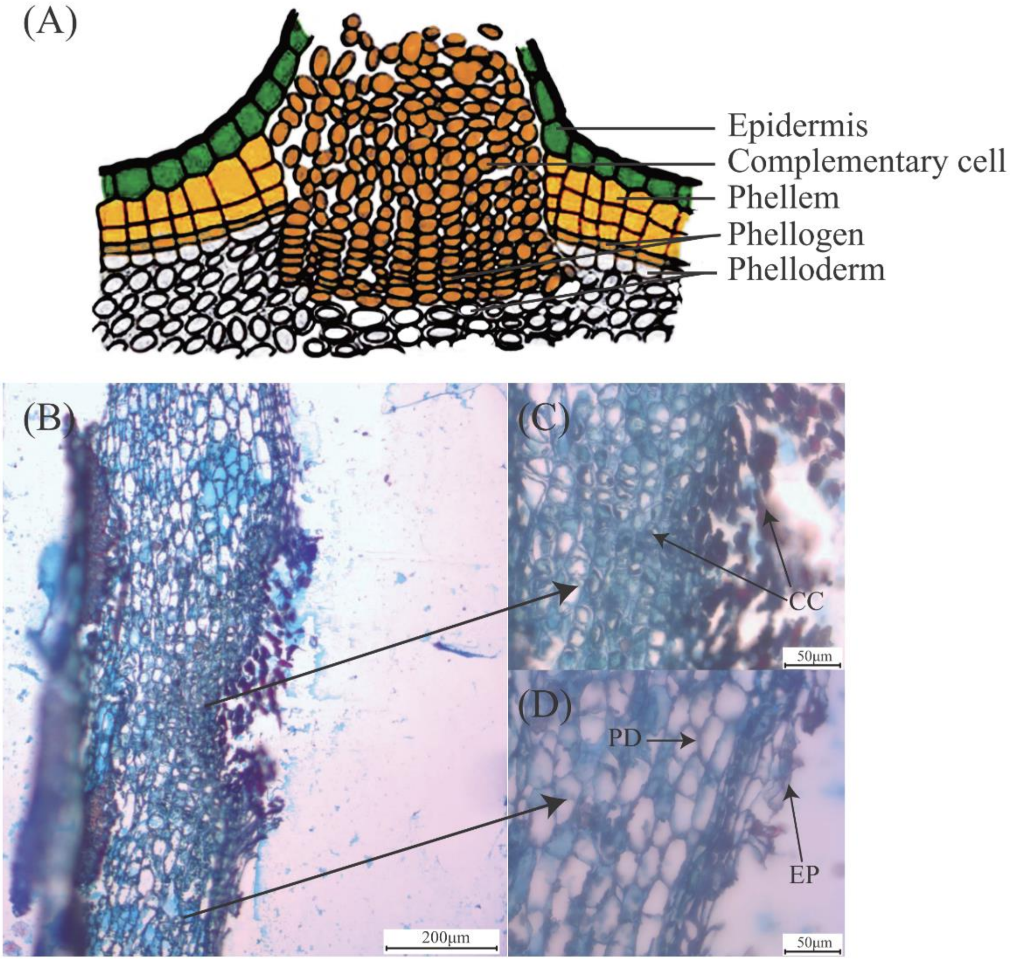


**Figure S1.** Characterization of lenticels in *Populus tomentosa*. **(A)** Graphic representation of lenticels in *P. tomentosa*. Green, yellow, brown, and colorless areas represent epidermis, phellogen, filling tissue, and phelloderm, respectively. **(B-D)** Transverse sections of young stems of *P. tomentosa.* CC, complementary cells; PD, phelloderm; EP, epidermis. Bars = 200μm (b); Bars = 200μm (b,c).

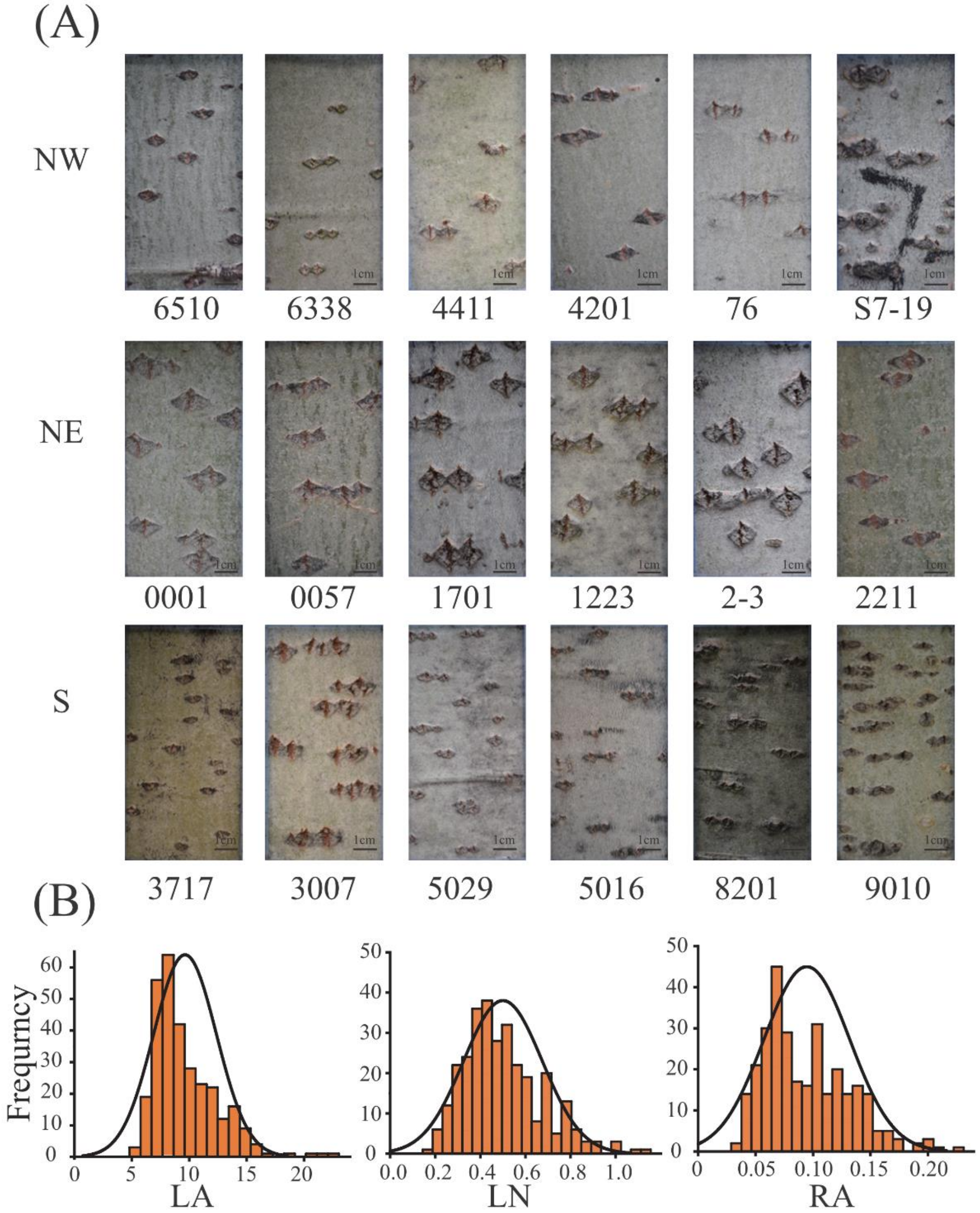


**Figure S2.** Phenotypic variations in the lenticels of the *Populus tomentosa* population. **(A)** Lenticel phenotypic characteristics in the three subpopulations. NW, NE, and S represent the northwest, northeast, and southern subpopulations, respectively. Numbers below the images represent the numbers of individuals with different genotypes. Bars = 1cm. **(B)** Histogram of lenticel area (LA), lenticel number (LN), and the ratio of the total LA to the area of a standard rectangular sampling window (RA).

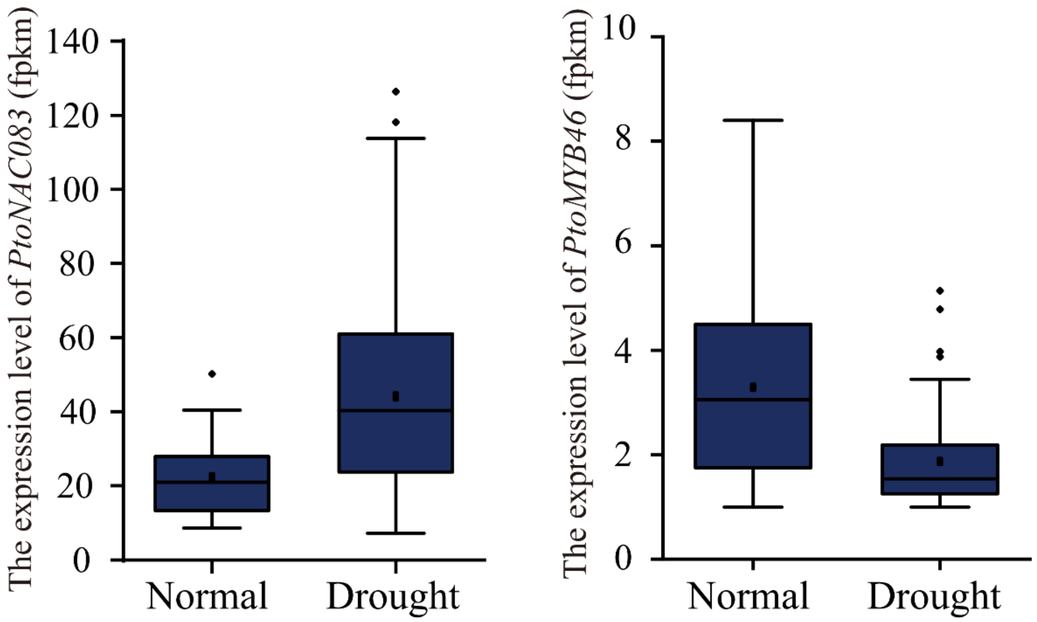


**Figure S3** The expression of *PtoNAC083* and *PtoMYB46* under drought and normal conditions in a population constituted with 100 accessions of *P. tomentosa*. FPKM: Fragments Per Kilobase of exon model per Million mapped fragments.

## 2 Supporting Tables:

**Table S1** Statistical analysis of three lenticel traits of *Populus tomentosa.*

| Trait | Min | Max | Mean | SD | SK | CV (%) | Clonal repeatability (P-value) |
|---|---|---|---|---|---|---|---|
| **LA** | 0.1875 | 1.15 | 0.4805 | 0.169 | 1.037 | 35.17 | 0.467(4.86E-40) |
| **LN** | 5.25 | 25 | 9.47 | 2.4808 | 1.067 | 26.2 | 0.549(4.67E-56) |
| **RA** | 0.03115 | 0.2098 | 0.0897 | 0.0355 | 0.91 | 39.58 | 0.515(8.67E-49) |

SD, SK and CV represent standard deviation, skewedness and coefficient of variation, respectively; LA, LN and RA represent single lenticel area, lenticel number, ratio of the total lenticel area and total area of the rectangular, respectively. Clonal repeatability represent repeatability estimation for lenticel-related traits.

**Table S2** Correlations of lenticel area (LA), lenticel number (LN), and the ratio of the total LA to the area of a standard rectangular sampling window (RA) with the diameter at breast height (DBH) of *Populus tomentosa*.

| Phenotype | LA | LN | RA | DBH |
|---|---|---|---|---|
| **LA** | 1 | -0.149* | 0.775** | 0.151* |
| **LN** | -0.149* | 1 | 0.481** | -0.043 |
| **RA** | 0.770** | 0.475** | 1 | 0.114 |
| **DBH** | 0.153* | -0.121* | 0.066 | 1 |

Data above the diagonal represent Pearson correlation coefficients; data below the diagonal represent Spearman correlation coefficients. * and ** represent significant correlation at Po0.05 and Po0.01; DBH represent diameter at breast height.

**Table S3** Analysis of variance of phenotypic variation in the three subpopulations.

| Phenotype | Group mean | | Sum of squares between groups | Within groups sum of squares | F | P |
|---|---|---|---|---|---|---|
| | NW | 0.4459 | | | | |
| **LA** | NE | 0.5596 | 1.0642175 | 7.562118219 | 21.10951195 | 2.64585E-09 |
| | S | 0.4284 | | | | |
| | NW | 7.856 | | | | |
| **LN** | NE | 8.8495 | 765.1189743 | 1093.543214 | 104.9504443 | 2.78819E-35 |
| | S | 11.637 | | | | |
| | NW | 0.0698 | | | | |
| **RA** | NE | 0.0979 | 0.053191371 | 0.327033647 | 24.39720098 | 1.52322E-10 |
| | S | 0.0991 | | | | |

Data from one-way analysis of variance; F represent ratio of squared deviation to degree of freedom between groups and groups; P represent the level of significance.

**Table S4** Multiple comparisons of phenotype data from different subpopulations.

| Phenotype | Subpopulation comparison | Mean difference | Standard error | Statistical significance |
|---|---|---|---|---|
| | NW-NE | -.113667793* | 0.022459795 | 7.28727E-07 |
| **LA** | NE-S | .131245497* | 0.021920921 | 6.0891E-09 |
| | NW-S | 0.010334722 | 0.022048723 | 0.639608768 |
| | NW-NE | -1.019791876* | 0.27008597 | 0.000192265 |
| **LN** | NE-S | -2.774645643* | 0.263605842 | 2.94303E-22 |
| | NW-S | -3.794437519* | 0.273736741 | 4.16185E-34 |
| | NW-NE | -.028106264* | 0.004670682 | 5.15256E-09 |
| **RA** | NE-S | 0.001233654 | 0.004558619 | 0.786869292 |
| | NW-S | -.029339918* | 0.004733816 | 1.88691E-09 |

Back Testing with Least-Significant Difference (LSD); * represent a significant difference in the mean value of 0.001

**Table S5** Climate factors in the three subgroups.

| Climatic factors | S | NE | NW |
|---|---|---|---|
| **Latitude(°N)** | 34.22 | 38.18 | 36.33 |
| **Longitude(°E)** | 113.2 | 116.43 | 110.2 |
| **Altitude(m)** | 224 | 66 | 1098 |
| **Average temperature in January(℃)** | -0.43 | -4.2 | -5.7 |
| **Average temperature in July(℃)** | 27 | 26.4 | 21.5 |
| **Drought index** | 0.65 | 0.94 | 0.7 |
| **Relative humidity(%)** | 69 | 62 | 58 |
| **Annual average precipitation(mm)** | 733 | 606 | 544 |
| **Annual average temperature(℃)** | 18.9 | 12.2 | 8.84 |

**Table S6** Full details of genome-wide association studies (GWAS) of *Populus tomentosa* lenticels, indicating single nucleotide (SNP) information, associated *P* values, additive and dominant effects, marker $r^2$ values, and *Arabidopsis* homolog and putative gene functions.

| Trait | SNP | Alleles | Location | Add_effect | Dom_effect | Marker_R^2 | SNP P-value | Populus tomentosa gene | ATG(Arabidopsis thaliana gene) | ATG Synonyms (abbreviation) in Plants |
|---|---|---|---|---|---|---|---|---|---|---|
| LA | Chr3_4043365 | T/C | Promoter | -0.1568753 | -0.1110234 | 0.10964 | 5.41161E-10 | Ptom.003G.00501 | AT5G13180 | NAC domain containing protein 83 |
| LA | Chr10_14339085 | A/G | Downstream | 1.5682021 | | 0.11466 | 1.02598E-09 | Ptom.010G.01976 | | |
| LA | Chr18_5120279 | G/A | Upstream | 0.0220467 | 1.1403763 | 0.10487 | 1.13789E-09 | Ptom.018G.00494 | | |
| LA | Chr17_5587165 | T/C | Promoter | 0.4848954 | | 0.09531 | 1.99247E-09 | Ptom.017G.00527 | AT3G60470 | Plant protein of unknown function (DUF247) |
| LA | Chr5_436708 | C/T | Promoter | -0.2249489 | 1.1033446 | 0.05884 | 3.38914E-09 | Ptom.005G.00059 | AT1G56600 | galactinol synthase 2 |
| LA | Chr14_5078112 | C/T | Exon | -0.184364 | | 0.15034 | 3.76948E-09 | Ptom.014G.00612 | AT3G61870 | |
| | | | | | | | | Ptom.014G.00614 | | |
| LA | Chr14_14227167 | G/A | Null | 0.4339485 | -1.2663843 | 0.05267 | 4.44018E-09 | null | | |
| LA | Chr19_12498058 | C/T | Intron | | -1.4551093 | 0.05865 | 4.54231E-09 | Ptom.019G.00696 | AT1G58807 | Disease resistance protein (CC-NBS-LRR class) family |
| LA | Chr2_16529660 | T/G | Downstream | 0.4561759 | | 0.07527 | 4.70312E-09 | Ptom.002G.02153 | AT1G02730 | cellulose synthase-like D5 |
| LA | Chr17_12646067 | C/G | Null | | -1.3460867 | 0.11273 | 5.04937E-09 | null | | |
| LA | Chr1_738204 | T/C | Intron | -0.9194695 | -1.6509011 | 0.06893 | 5.76134E-09 | Ptom.001G.00083 | | |
| | | | | | | | | Ptom.001G.00082 | AT3G13540 | myb domain protein 5 |
| LA | Chr11_12411443 | A/T | Intron | 0.3225828 | 1.1326206 | 0.0753 | 6.31763E-09 | Ptom.011G.00779 | AT1G16970 | KU70 homolog |
| LA | Chr1_49752248 | A/T | Null | 0.3702983 | | 0.06613 | 6.58464E-09 | null | | |
| LA | Chr15_12882962 | G/A | Null | | -0.5530657 | 0.13786 | 6.67854E-09 | null | | |
| LA | Chr3_1314080 | C/T | Upstream | -0.1551254 | 1.8817363 | 0.09815 | 7.74739E-09 | Ptom.003G.00171 | AT3G63530 | RING/U-box superfamily protein |
| LA | Chr6_2874331 | A/G | Null | | | 0.08267 | 8.43841E-09 | null | | |
| LA | Chr5_2053022 | C/T | Promoter | 0.8416913 | | 0.09634 | 9.27739E-09 | Ptom.005G.00279 | AT3G05200 | RING/U-box superfamily protein |

| | | | | | | | | | | |
|---|---|---|---|---|---|---|---|---|---|---|
| LA | Chr9_3551378 | G/A | Upstream | -0.7816313 | -0.2070018 | 0.08679 | 1.05637E-08 | Ptom.009G.00386 | AT5G12870 | myb domain protein 46 |
| LA | Chr2_9795019 | C/T | Downstream | 0.1451829 | | 0.11861 | 1.06292E-08 | Ptom.002G.01412 | AT4G17650 | Polyketide cyclase / dehydrase and lipid transport protein |
| | | | | | | | | Ptom.002G.01411 | | |
| LA | Chr3_5725284 | G/T | Downstream | 0.4450998 | -0.5660883 | 0.11207 | 1.28839E-08 | Ptom.003G.00730 | AT4G24040 | trehalase 1 |
| LA | Chr14_16339179 | C/T | Null | 1.1274179 | | 0.07924 | 1.354E-08 | null | | |
| LA | Chr10_13623192 | A/C | Exon | 0.89573 | | 0.08635 | 1.49898E-08 | Ptom.010G.01894 | AT3G25400 | |
| LA | Chr16_11377075 | C/T | Promoter | 0.0764206 | 1.4057267 | 0.15975 | 1.53742E-08 | Ptom.016G.00952 | AT5G01720 | RNI-like superfamily protein |
| LA | Chr7_3538903 | T/C | Null | | 1.1016426 | 0.09637 | 1.54136E-08 | null | | |
| LA | Chr13_4783422 | C/T | Null | -0.26536 | | 0.11383 | 2.13179E-08 | null | | |
| LA | Chr5_11035663 | A/T | Upstream | | -0.7559246 | 0.09349 | 2.17057E-08 | Ptom.005G.01280 | AT5G67080 | mitogen-activated protein kinase kinase kinase 19 |
| LA | Chr6_4862894 | A/T | Downstream | | -1.86179 | 0.10264 | 2.20664E-08 | Ptom.006G.00608 | AT3G15880 | WUS-interacting protein 2 |
| LA | Chr18_1140161 | A/T | Null | 1.2095517 | -1.2995198 | 0.061850469 | 2.23784E-08 | null | | |
| LA | Chr17_8965052 | A/G | Promoter | -1.161308 | | 0.07948 | 2.83511E-08 | Ptom.017G.00796 | AT1G04920 | sucrose phosphate synthase 3F |
| LA | Chr7_9751093 | A/G | Null | | 0.3439463 | 0.06641 | 3.06379E-08 | null | | |
| LA | Chr1_42858403 | C/T | Upstream | -0.6034272 | -0.4064774 | 0.07345 | 3.06991E-08 | Ptom.001G.03753 | AT3G15290 | 3-hydroxyacyl-CoA dehydrogenase family protein |
| | | | | | | | | Ptom.001G.03754 | | |
| LA | Chr4_12566175 | C/A | Upstream | | 2.734844 | | 3.29613E-08 | Ptom.004G.01250 | AT5G28040 | DNA-binding storekeeper protein-related transcriptional regulator |
| | | | | | | | | Ptom.004G.01251 | AT1G66345 | Pentatricopeptide repeat (PPR) superfamily protein |
| LA | Chr1_42858469 | T/C | Upstream | 1.1467652 | -1.6514203 | 0.03196 | 4.0637E-08 | Ptom.001G.03753 | AT3G15290 | 3-hydroxyacyl-CoA dehydrogenase family protein |
| | | | | | | | | Ptom.001G.03754 | | |
| LA | Chr4_4478576 | C/A | Exon | -1.4143488 | -1.1627538 | 0.09769 | 4.07124E-08 | Ptom.004G.00537 | AT3G29230 | Tetratricopeptide repeat (TPR)-like superfamily protein |
| | | | | | | | | Ptom.004G.00535 | AT4G38690 | PLC-like phosphodiesterases superfamily protein |
| LA | Chr19_15920988 | G/A | Promoter | -0.545629 | -0.4645084 | 0.12397 | 4.08356E-08 | Ptom.019G.00899 | AT3G05550 | Hypoxia-responsive family protein |

| | | | | | | | | | | |
|---|---|---|---|---|---|---|---|---|---|---|
| LA | Chr1_42858623 | C/T | Upstream | -0.5175407 | -0.492615 | 0.06893 | 4.66789E-08 | Ptom.001G.03753 | AT3G15290 | 3-hydroxyacyl-CoA dehydrogenase family protein |
| | | | | | | | | Ptom.001G.03754 | | |
| LA | Chr1_42859241 | T/C | Upstream | | 1.3087939 | 0.06182 | 5.06991E-08 | Ptom.001G.03753 | AT3G15290 | 3-hydroxyacyl-CoA dehydrogenase family protein |
| | | | | | | | | Ptom.001G.03754 | | |
| LA | Chr1_42860206 | C/T | Upstream | 0.5090495 | -1.1213413 | 0.06893 | 5.06991E-08 | Ptom.001G.03753 | AT3G15290 | 3-hydroxyacyl-CoA dehydrogenase family protein |
| | | | | | | | | Ptom.001G.03754 | | |
| LA | Chr7_9756305 | A/C | Five_prime_UTR | 1.1759367 | -0.8097464 | 0.07794 | 5.06991E-08 | Ptom.007G.00921 | AT4G33565 | RING/U-box superfamily protein |
| LA | Chr17_14295325 | C/T | Downstream | 0.5834254 | -0.5226717 | 0.06534 | 5.24568E-08 | Ptom.017G.01164 | | |
| | | | | | | | | Ptom.017G.01165 | | |
| LA | Chr17_5428225 | C/T | Downstream | 1.3676318 | 0.73132 | 0.11772 | 5.26673E-08 | Ptom.017G.00524 | AT3G60470 | Plant protein of unknown function (DUF247) |
| LA | Chr16_4330649 | | Upstream | 0.1106 | | 0.10014 | 4.17167E-08 | Ptom.016G.00430 | AT5G22300 | nitrilase 4 |
| LN | Chr7_10376607 | A/C | Intron | -0.39881 | -0.58428 | 0.09408 | 1.15327E-10 | Ptom.007G.00997 | AT1G05270 | TraB family protein |
| | | | | | | | | Ptom.007G.00998 | AT1G05270 | TraB family protein |
| LN | Chr16_14009977 | G/T | Null | -2.3772 | -1.6397 | 0.16296 | 1.10944E-09 | null | | |
| LN | Chr11_5464781 | A/G | Upstream | | 1.1826067 | 0.08224 | 1.11464E-09 | Ptom.011G.00380 | AT2G33460 | ROP-interactive CRIB motif-containing protein 1 |
| LN | Chr1_3852315 | C/T | Promoter | 1.47613 | | 0.06877 | 1.42183E-09 | Ptom.001G.00446 | AT2G02230 | phloem protein 2-B1 |
| LN | Chr1_9658400 | C/T | Downstream | | -1.29656 | 0.07431 | 1.80505E-09 | Ptom.001G.01079 | AT5G59090 | subtilase 4.12 |
| | | | | | | | | Ptom.001G.01078 | | |
| LN | Chr4_1772820 | A/G | Upstream | | 0.16942 | 0.16841 | 2.65135E-09 | Ptom.004G.00190 | AT5G60680 | Protein of unknown function, DUF584 |
| | | | | | | | | Ptom.004G.00191 | AT2G28410 | |
| LN | Chr5_14956223 | A/G | Downstream | 0.28947 | 1.1737 | 0.09304 | 2.70639E-09 | Ptom.005G.01540 | AT1G21722 | |
| LN | Chr6_13662334 | C/T | Downstream | 1.45167 | -2.24582 | 0.07018 | 3.09566E-09 | Ptom.006G.01508 | AT2G19930 | RNA-dependent RNA polymerase family protein |
| LN | Chr12_2136415 | T/C | Null | -3.14004 | | 0.07891 | 3.35276E-09 | null | | |
| LN | Chr18_4898061 | A/C | Null | 2.89401 | -0.23419 | 0.11105 | 3.51972E-09 | null | | |
| LN | Chr9_9711494 | T/C | Intron | 0.25673 | 0.99834 | 0.07613 | 4.40561E-09 | Ptom.009G.01338 | | |
| | | | | | | | | Ptom.009G.01337 | AT2G29130 | laccase 2 |

| | | | | | | | | | | |
|---|---|---|---|---|---|---|---|---|---|---|
| LN | Chr9_1553835 | T/C | Null | 0.26998 | 1.67978 | 0.07384 | 4.7886E-09 | null | | |
| LN | Chr19_8584383 | C/T | Upstream | 0.14993 | | 0.09556 | 5.02033E-09 | Ptom.019G.00523 | | |
| LN | Chr2_21127271 | T/C | Downstream | 0.4873099 | | 0.11347 | 6.03142E-09 | Ptom.002G.02464 | AT1G04960 | Protein of unknown function (DUF1664) |
| | | | | | | | | Ptom.002G.02463 | AT2G04520 | Nucleic acid-binding, OB-fold-like protein |
| LN | Chr11_5827356 | T/A | Null | 0.6128911 | -3.8345 | 0.07459 | 6.14803E-09 | null | | |
| LN | Chr4_13568631 | T/A | Null | -1.0098254 | -0.37762 | 0.16029 | 6.60146E-09 | null | | |
| LN | Chr13_7469817 | T/C | Downstream | 1.4282968 | -0.65729 | 0.07663 | 6.74032E-09 | Ptom.013G.00600 | | |
| | | | | | | | | Ptom.013G.00601 | AT5G16730 | Plant protein of unknown function (DUF827) |
| LN | Chr3_2653020 | A/G | Null | -1.91779 | -0.32925 | 0.09769 | 6.99506E-09 | null | | |
| LN | Chr17_4717361 | A/G | Upstream | -0.36758 | | 0.04293 | 7.59074E-09 | Ptom.017G.00466 | AT1G80350 | P-loop containing nucleoside triphosphate hydrolases superfamily protein |
| LN | Chr14_1028654 | T/C | Upstream | | -0.41781 | 0.10666 | 8.70433E-09 | Ptom.014G.00137 | AT5G43060 | Granulin repeat cysteine protease family protein |
| LN | Chr2_8900384 | G/A | Promoter | 5.54479 | -2.0843 | 0.1124 | 8.82431E-09 | Ptom.002G.01294 | AT3G49940 | LOB domain-containing protein 38 |
| | | | | | | | | Ptom.002G.01295 | AT1G49890 | Family of unknown function (DUF566) |
| LN | Chr2_8900427 | T/G | Promoter | -0.26461 | 0.86415 | 0.09665 | 9.82431E-09 | Ptom.002G.01294 | AT3G49940 | LOB domain-containing protein 38 |
| | | | | | | | | Ptom.002G.01295 | AT1G49890 | Family of unknown function (DUF566) |
| LN | Chr17_1435896 | G/A | Null | 1.552 | | 0.17896 | 9.19582E-09 | null | | |
| LN | Chr18_10221057 | T/A | Downstream | -2.0677 | -1.5575 | 0.10766 | 9.93135E-09 | Ptom.018G.01078 | AT5G57510 | |
| | | | | | | | | Ptom.018G.01078 | AT5G57510 | |
| LN | Chr14_3043493 | T/C | Downstream | | -0.58501 | 0.09101 | 1.01926E-08 | Ptom.014G.00353 | AT1G05490 | chromatin remodeling 31 |
| | | | | | | | | Ptom.014G.00352 | AT2G44745 | WRKY family transcription factor |
| LN | Chr18_8755947 | T/C | Null | -0.97397 | -4.0805 | 0.15585 | 1.05312E-08 | null | | |
| LN | Chr18_6252711 | A/G | Intron | 0.30323 | 1.379177 | 0.09769 | 1.14423E-08 | Ptom.018G.00629 | AT2G19080 | metaxin-related |
| LN | Chr16_14011677 | G/A | Null | -0.25768 | -1.75366 | 0.12715 | 1.23169E-08 | null | | |
| LN | Chr6_6559040 | A/T | Promoter | | -0.93574 | 0.11771 | 1.37452E-08 | Ptom.006G.00835 | AT3G14730 | Pentatricopeptide repeat (PPR) superfamily protein |
| | | | | | | | | Ptom.006G.00836 | AT3G53900 | uracil phosphoribosyltransferase |

| | | | | | | | | | | |
|---|---|---|---|---|---|---|---|---|---|---|
| LN | Chr17_9336158 | G/A | gene | -4.8607 | -1.5294 | 0.13724 | 1.4163E-08 | Ptom.017G.00825 | | |
| LN | Chr5_11449913 | A/G | Promoter | 0.56974 | | 0.09769 | 1.63401E-08 | Ptom.005G.01313 | AT4G37270 | heavy metal atpase 1 |
| | | | | | | | | Ptom.005G.01312 | AT4G37260 | myb domain protein 73 |
| LN | Chr3_1558853 | T/A | Downstream | -0.89761 | -0.33102 | 0.09786 | 1.64274E-08 | Ptom.003G.00199 | | |
| LN | Chr1_10992344 | T/G | Null | -3.76271 | -0.84357 | 0.10964 | 1.70872E-08 | null | | |
| LN | Chr1_16727590 | A/C | Null | | -0.81317 | 0.08632 | 1.83907E-08 | null | | |
| LN | Chr18_10171116 | C/T | Downstream | -2.3446 | -1.312 | 0.1135 | 2.08129E-08 | Ptom.018G.01072 | AT4G25830 | Uncharacterised protein family (UPF0497) |
| | | | | | | | | Ptom.018G.01071 | AT5G56080 | nicotianamine synthase 2 |
| LN | Chr5_14460934 | G/A | Null | 0.847965 | | 0.09696 | 2.19618E-08 | null | | |
| LN | Chr6_5854014 | A/C | Promoter | 0.65235 | 0.01839 | 0.07959 | 2.57959E-08 | Ptom.006G.00759 | AT3G52750 | Tubulin/FtsZ family protein |
| | | | | | | | | Ptom.006G.00758 | AT2G36270 | Basic-leucine zipper (bZIP) transcription factor family protein |
| LN | Chr1_39951663 | G/T | Null | 0.73474 | -1.23099 | 0.06877 | 2.65632E-08 | null | | |
| LN | Chr10_7228249 | A/T | Intron | | -0.49648 | 0.06893 | 2.67429E-08 | Ptom.010G.01098 | AT2G26710 | Cytochrome P450 superfamily protein |
| LN | Chr6_15199103 | C/T | Upstream | 1.47607 | -0.73188 | 0.09284 | 2.74951E-08 | Ptom.006G.01598 | AT5G54010 | UDP-Glycosyltransferase superfamily protein |
| LN | Chr5_14956207 | C/T | Downstream | -3.6819 | -2.3214 | 0.09892 | 2.75799E-08 | Ptom.005G.01540 | AT1G21722 | |
| LN | Chr4_13568629 | G/A | Null | 0.56192 | -0.56017 | 0.09769 | 2.80169E-08 | null | | |
| LN | Chr2_21127255 | T/G | Downstream | 0.54442 | 2.42554 | 0.02668 | 3.27752E-08 | Ptom.002G.02464 | AT1G04960 | Protein of unknown function (DUF1664) |
| | | | | | | | | Ptom.002G.02463 | AT2G04520 | Nucleic acid-binding, OB-fold-like protein |
| LN | Chr2_21127290 | G/A | Downstream | 0.792219 | 0.36013 | 0.09956 | 3.29558E-08 | Ptom.002G.02464 | AT1G04960 | Protein of unknown function (DUF1664) |
| | | | | | | | | Ptom.002G.02463 | AT2G04520 | Nucleic acid-binding, OB-fold-like protein |
| LN | Chr18_4898066 | C/A | Null | 0.39298 | | 0.05914 | 4.55657E-08 | null | | |
| LN | Chr16_14677459 | C/T | Intron | -0.07807 | | 0.09551 | 3.69246E-08 | Ptom.016G.01280 | AT2G32170 | S-adenosyl-L-methionine-dependent methyltransferases superfamily protein |
| LN | Chr19_9844474 | T/C | Null | | 0.95766 | 0.10577 | 3.91516E-08 | null | | |
| LN | Chr1_14025085 | G/A | Downstream | -0.84623 | -0.95757 | 0.07849 | 4.23501E-08 | Ptom.001G.01548 | AT1G17260 | autoinhibited H(+)-ATPase isoform 10 |

| | | | | | | | | | | |
|---|---|---|---|---|---|---|---|---|---|---|
| LN | Chr7_11736920 | A/G | Null | | -0.10199 | 0.08697 | 4.42534E-08 | null | | |
| LN | Chr7_4680175 | G/A | Null | 0.06232 | -0.53849 | 0.08129 | 4.46487E-08 | null | | |
| LN | Chr16_10727563 | A/G | Upstream | 2.69652 | -1.8806 | 0.14351 | 4.59711E-08 | Ptom.016G.00892 | | |
| LN | Chr2_18582313 | C/G | Promoter | 0.21261 | | | 4.69554E-08 | Ptom.002G.02263 | AT3G63000 | NPL4-like protein 1 |
| LN | Chr1_3027784 | C/T | Downstream | 0.01083 | -0.19609 | 0.07055 | 4.84415E-08 | Ptom.001G.00367 | | |
| RA | Chr5_15162313 | G/A | Downstream | | -0.06944 | 0.09193 | 2.47672E-09 | Ptom.005G.01558 | | |
| | | | | | | | | Ptom.005G.01557 | AT5G20240 | K-box region and MADS-box transcription factor family protein |
| RA | Chr3_4043365 | T/C | Promoter | 0.045838 | 0.068166 | 0.09604 | 4.18145E-09 | Ptom.003G.00501 | AT5G13180 | NAC domain containing protein 83 |
| RA | Chr11_12601096 | T/C | Downstream | 0.092375 | | 0.07668 | 8.10177E-09 | Ptom.011G.00792 | AT1G78800 | UDP-Glycosyltransferase superfamily protein |
| | | | | | | | | Ptom.011G.00791 | AT1G78780 | pathogenesis-related family protein |
| RA | Chr5_15162302 | T/G | Downstream | | 0.083482 | 0.07088 | 9.83671E-09 | Ptom.005G.01558 | | |
| | | | | | | | | Ptom.005G.01557 | AT5G20240 | K-box region and MADS-box transcription factor family protein |
| RA | Chr16_13337341 | G/C | Intron | -0.49695 | | 0.09171 | 1.16047E-08 | Ptom.016G.01184 | AT5G47540 | Mo25 family protein |
| RA | Chr1_17763020 | C/T | Null | 0.097846 | -0.05347 | 0.07566 | 1.67616E-08 | null | | |
| RA | Chr11_12601097 | G/A | Upstream | -1.02705 | | 0.10423 | 2.0251E-08 | Ptom.011G.00791 | AT1G78780 | pathogenesis-related family protein |
| | | | | | | | | Ptom.011G.00792 | AT1G78800 | UDP-Glycosyltransferase superfamily protein |
| RA | Chr16_2934808 | G/A | Intron | -0.023479 | 0.043822 | 0.12637 | 2.24411E-08 | Ptom.016G.00300 | AT5G06680 | spindle pole body component 98 |
| | | | | | | | | Ptom.016G.00299 | | |
| RA | Chr14_5078112 | C/T | Exon | -0.16857 | -0.085927 | 0.13649 | 2.54129E-08 | Ptom.014G.00612 | AT3G61870 | |
| | | | | | | | | Ptom.014G.00614 | | |
| RA | Chr5_11035663 | A/T | Upstream | | -1.520954 | 0.09769 | 3.17383E-08 | Ptom.005G.01280 | AT5G67080 | mitogen-activated protein kinase kinase kinase 19 |
| RA | Chr2_16529613 | C/T | Downstream | -0.15322 | -0.05471 | 0.16756 | 3.22066E-08 | Ptom.002G.02153 | AT1G02730 | cellulose synthase-like D5 |
| RA | Chr2_7534451 | T/G | Upstream | 0.01792 | | 0.13489 | 3.65365E-08 | Ptom.002G.01132 | AT2G22500 | uncoupling protein 5 |
| RA | Chr2_16529660 | T/G | Downstream | -0.034835 | 0.28143 | 0.0676 | 3.77946E-08 | Ptom.002G.02153 | AT1G02730 | cellulose synthase-like D5 |

| | | | | | | | | | | |
|---|---|---|---|---|---|---|---|---|---|---|
| RA | Chr11_12601092 | A/G | Downstream | 0.11835 | -0.06676 | 0.07101 | 4.40993E-08 | Ptom.011G.00792 | AT1G78800 | UDP-Glycosyltransferase superfamily protein |
| | | | | | | | | Ptom.011G.00791 | AT1G78780 | pathogenesis-related family protein |
| RA | Chr11_12601086 | G/T | Downstream | | -0.134758 | 0.07412 | 4.58487E-08 | Ptom.011G.00792 | AT1G78800 | UDP-Glycosyltransferase superfamily protein |
| | | | | | | | | Ptom.011G.00791 | AT1G78780 | pathogenesis-related family protein |
| RA | Chr1_24209894 | A/G | Upstream | 0.6772771 | | 0.098627 | 5.041E-08 | Ptom.001G.02163 | AT3G11440 | myb domain protein 65 |
| | | | | | | | | Ptom.001G.02164 | AT5G56200 | C2H2 type zinc finger transcription factor family |
| RA | Chr9_3396423 | T/A | Promoter | -0.05366 | -0.031837 | 0.07233 | 5.09636E-08 | Ptom.009G.00359 | AT3G21070 | NAD kinase 1 |

**Table S7 Gene ontology (GO) analysis of 88 genes in genome-wide association studies (GWAS)**

| ID | Class | Description | *P*_value |
|---|---|---|---|
| GO:0018130 | Biological Process | heterocycle biosynthetic process | 0.000766907 |
| GO:1901362 | Biological Process | organic cyclic compound biosynthetic process | 0.000970141 |
| GO:0044271 | Biological Process | cellular nitrogen compound biosynthetic process | 0.001893394 |
| GO:0034654 | Biological Process | nucleobase-containing compound biosynthetic process | 0.002277556 |
| GO:0009737 | Biological Process | response to abscisic acid | 0.00329069 |
| GO:0044249 | Biological Process | cellular biosynthetic process | 0.004859225 |
| GO:1901576 | Biological Process | organic substance biosynthetic process | 0.005722752 |
| GO:0000271 | Biological Process | polysaccharide biosynthetic process | 0.005831 |
| GO:0033692 | Biological Process | cellular polysaccharide biosynthetic process | 0.005977 |
| GO:0030243 | Biological Process | cellulose metabolic process | 0.006054208 |
| GO:0030244 | Biological Process | cellulose biosynthetic process | 0.006054208 |
| GO:2001141 | Biological Process | regulation of RNA biosynthetic process | 0.006058442 |
| GO:1901360 | Biological Process | organic cyclic compound metabolic process | 0.00719034 |
| GO:0034641 | Biological Process | cellular nitrogen compound metabolic process | 0.007945358 |
| GO:0009058 | Biological Process | biosynthetic process | 0.007990306 |
| GO:0072351 | Biological Process | tricarboxylic acid biosynthetic process | 0.008064475 |
| GO:0009143 | Biological Process | nucleoside triphosphate catabolic process | 0.008064475 |
| GO:0006355 | Biological Process | regulation of transcription, DNA-templated | 0.008064475 |
| GO:0044281 | Biological Process | small molecule metabolic process | 0.009859495 |
| GO:0015927 | Molecular Function | trehalase activity | 0.006413559 |
| GO:0042162 | Molecular Function | telomeric DNA binding | 0.006413559 |
| GO:0004555 | Molecular Function | alpha,alpha-trehalase activity | 0.006413559 |
| GO:0008194 | Molecular Function | UDP-glycosyltransferase activity | 0.008542535 |
| GO:0016759 | Molecular Function | cellulose synthase activity | 0.009106671 |
| GO:0016760 | Molecular Function | cellulose synthase (UDP-forming) activity | 0.009106671 |

**Table S8** Detailed information on significant epistatic SNP pairs for lenticel traits from the GWAS results for *Populus tomentosa*.

| **Locus1** | **Locus2** | **Trait** | **Test** | **Effect** | ***P*_value** |
|---|---|---|---|---|---|
| Chr11_12601086 | Chr1_24209894 | RA | DA | 1.21 | 4.43E-05 |
| Chr5_11449913 | Chr3_4043365 | LN | AD | 2.28 | 5.70E-05 |
| Chr1_16727590 | Chr4_13568629 | LN | DA | 4.27 | 1.20E-04 |
| Chr1_24209894 | Chr7_4680175 | RA | AA | -0.04 | 1.21E-04 |
| Chr6_6559040 | Chr3_4043365 | RA | DD | -0.12 | 1.31E-04 |
| Chr1_738204 | Chr17_8965052 | LN | DA | -3.71 | 1.99E-04 |
| Chr17_5587165 | Chr5_15162302 | LN | DA | -6.24 | 2.03E-04 |
| Chr1_738204 | Chr3_4043365 | RA | AA | 0.14 | 2.30E-04 |
| Chr3_4043365 | Chr9_3551378 | RA | AA | 0.05 | 5.74E-04 |
| Chr2_16529660 | Chr17_4717361 | LA | AA | 1.81 | 8.74E-04 |
| Chr2_16529660 | Chr18_4898061 | LN | DD | -1.91 | 8.96E-04 |
| Chr3_4043365 | Chr11_12601086 | LA | AD | 1.76 | 8.99E-04 |
| Chr7_9756305 | Chr9_3551378 | LA | AA | -1.34 | 9.76E-04 |
| Chr7_3538903 | Chr13_4783422 | LA | DA | -0.81 | 3.61E-04 |
| Chr19_15920988 | Chr1_24209894 | LA | AA | -1.97 | 1.01E-03 |

**Table S9** Expression data for several genes detected by GWAS in different tissues of *Populus*.

| Gene model | Bark | Mature leaf | Mature xylem | Development leaf | Cambium | Immature xylem | Shoot apical meristem | Phloem | Root |
|---|---|---|---|---|---|---|---|---|---|
| Ptom.003G.00501 | 131.154 | 45.5445 | 249.25843 | 58.810133 | 11.0789 | 16.67136667 | 41.75576667 | 154.2964 | 142.9067 |
| Ptom.010G.01976 | 0 | 0.1661 | 0 | 0.5049927 | 0.53386 | 0.348659 | 0.210053667 | 3.303017 | 1.128293 |
| Ptom.017G.00527 | 3.20012 | 0.02392 | 0.5435447 | 0.0453617 | 1.193703 | 0.834944333 | 0.590132133 | 1.214697 | 14.26737 |
| Ptom.005G.00059 | 0 | 0 | 0 | 0 | 0.045888 | 0 | 0.141327833 | 0.058058 | 0.397954 |
| Ptom.014G.00612 | 86.8581 | 189.09 | 2.6535167 | 228.376 | 9.78194 | 9.20131 | 31.4143 | 7.159507 | 7.481237 |
| Ptom.014G.00614 | 0 | 3.25801 | 0.0200798 | 8.4079067 | 0.448124 | 0.503048 | 1.362269333 | 21.83177 | 14.64074 |
| Ptom.002G.02153 | 0.0571 | 0.05448 | 0.0754992 | 0.3471737 | 3.26614 | 1.308733 | 2.221064 | 0.79898 | 0.348697 |
| Ptom.001G.00082 | 7.40729 | 8.792 | 0.0507253 | 15.166567 | 0.025741 | 0.106235667 | 9.82374 | 3.081307 | 3.329467 |
| Ptom.011G.00779 | 10.7113 | 9.6928 | 9.9640933 | 9.22704 | 7.09809 | 5.857783333 | 15.5425 | 11.4163 | 11.06843 |
| Ptom.003G.00171 | 2.74461 | 2.18021 | 5.4929767 | 7.1723267 | 27.61305 | 18.57166667 | 11.69676667 | 25.1342 | 13.6794 |
| Ptom.005G.00279 | 2.80996 | 2.45449 | 37.5686 | 1.295016 | 49.8983 | 49.4059 | 6.042126667 | 116.8579 | 111.8777 |
| Ptom.009G.00386 | 0.065498 | 2.09758 | 5.7909667 | 2.1784167 | 18.00945 | 21.8945 | 2.553283333 | 0.34762 | 7.388103 |
| Ptom.002G.01412 | 5.35658 | 9.37143 | 9.8162133 | 9.4440333 | 6.749665 | 8.807696667 | 7.78124 | 8.485753 | 7.95188 |
| Ptom.002G.01411 | 31.819 | 41.8404 | 35.949033 | 24.2555 | 11.66295 | 11.62721 | 0.472033333 | 26.78183 | 15.01257 |
| Ptom.003G.00730 | 0.103294 | 0.07187 | 0.0271799 | 0.1454035 | 0 | 0.100731333 | 0.064168333 | 0.16578 | 0.108869 |
| Ptom.010G.01894 | 0 | 0.1013 | 0 | 0.0426333 | 0 | 0 | 0.158526667 | 1.532783 | 0.170544 |
| Ptom.016G.00952 | 2.65851 | 8.04108 | 4.6599533 | 5.69669 | 2.28168 | 3.19342 | 9.018703333 | 4.803523 | 7.142123 |
| Ptom.005G.01280 | 0.390556 | 5.95875 | 63.195467 | 3.4277067 | 10.99736 | 34.09805667 | 3.465086667 | 207.4563 | 111.1938 |
| Ptom.006G.00608 | 29.0105 | 23.4421 | 43.189733 | 32.372167 | 20.2088 | 23.6476 | 34.95586667 | 63.5911 | 47.49133 |
| Ptom.017G.00796 | 0.020296 | 23.7742 | 1.2527027 | 20.2964 | 0.267587 | 0.236191467 | 0.235798967 | 0.374267 | 0.434189 |
| Ptom.001G.03753 | 21.3178 | 20.5403 | 14.477433 | 15.197167 | 23.89615 | 20.15906667 | 16.39766667 | 13.16407 | 21.533 |

| | | | | | | | | | |
|---|---|---|---|---|---|---|---|---|---|
| Ptom.001G.03754 | 0 | 0 | 0 | 0 | 0 | 0 | 0 | 0 | 0 |
| Ptom.004G.01250 | 2.38795 | 4.10355 | 5.3919 | 3.52847 | 2.93877 | 3.684846667 | 4.045636667 | 2.673453 | 2.687983 |
| Ptom.004G.01251 | 9.81575 | 3.24426 | 5.5955167 | 2.90514 | 2.84269 | 3.312183333 | 3.667803333 | 4.814787 | 3.126393 |
| Ptom.004G.00537 | 3.01316 | 4.30284 | 2.61023 | 5.1809133 | 0.864999 | 1.124666333 | 1.577 | 1.32672 | 1.54329 |
| Ptom.004G.00535 | 14.7962 | 16.6119 | 137.2371 | 24.5038 | 92.03935 | 97.77756667 | 20.6903 | 10.67598 | 46.26223 |
| Ptom.019G.00899 | 0 | 0 | 0.0980483 | 0.1798383 | 0 | 1.030483333 | 0.225639 | 0 | 0.085994 |
| Ptom.007G.00921 | 1.44241 | 0.29952 | 5.2764 | 0.365566 | 0.362215 | 0.767255 | 0.895365667 | 0.888716 | 3.219043 |
| Ptom.017G.01164 | 0 | 0 | 0 | 0.0534783 | 0.064562 | 0 | 0 | 0.09713 | 0 |
| Ptom.017G.01165 | 0 | 0 | 0 | 0 | 0 | 0 | 0 | 0 | 0 |
| Ptom.017G.00524 | 1.15818 | 0.0282 | 0 | 0.121806 | 0.260235 | 0.505930667 | 0.2301466 | 1.697373 | 1.368813 |
| Ptom.007G.00997 | 8.66463 | 6.89081 | 5.53602 | 6.95798 | 10.2249 | 8.29363 | 9.283536667 | 9.703787 | 7.243357 |
| Ptom.007G.00998 | 20.4152 | 14.1158 | 7.0488033 | 10.625277 | 11.04849 | 10.26762667 | 16.84276667 | 8.15326 | 10.73666 |
| Ptom.011G.00380 | 0 | 0.471 | 0 | 1.025938 | 0 | 0 | 0.118647933 | 0.030185 | 0.026536 |
| Ptom.001G.00446 | 2.52853 | 1.29748 | 4.66426 | 1.1507227 | 2.476905 | 1.723236667 | 1.762494333 | 6.95875 | 5.00782 |
| Ptom.001G.01079 | 0 | 0 | 0 | 0 | 0 | 0 | 0 | 0 | 0 |
| Ptom.001G.01078 | 4.21656 | 8.72491 | 6.3637967 | 6.0974067 | 2.737686 | 2.009487 | 12.5637 | 4.631253 | 12.33113 |
| Ptom.004G.00190 | 4.7193 | 7.93445 | 26.1796 | 7.4344633 | 7.80097 | 8.056393333 | 6.04716 | 4.985363 | 10.3769 |
| Ptom.004G.00191 | 0 | 0.11605 | 1.9745333 | 0.3274517 | 14.1053 | 12.13953 | 0.1262173 | 0.065646 | 0.843429 |
| Ptom.005G.01540 | 0 | 0 | 0 | 0 | 0 | 0 | 0 | 0 | 0 |
| Ptom.006G.01508 | 0.574935 | 0.29654 | 0.6782317 | 0.4719037 | 0.979677 | 0.760059333 | 1.231866667 | 16.27744 | 5.09659 |
| Ptom.009G.01338 | 49.9177 | 174.57 | 0.3449827 | 196.652 | 1.544636 | 2.24405 | 24.8892 | 3.81529 | 0.909105 |
| Ptom.009G.01337 | 0.050649 | 0.25533 | 6.1765733 | 0.3662477 | 123.2102 | 140.5653333 | 0.037048 | 0.131958 | 5.33519 |
| Ptom.019G.00523 | 0 | 0 | 0 | 0 | 0 | 0 | 0 | 0 | 0 |
| Ptom.002G.02464 | 7.18146 | 7.029 | 9.16464 | 5.3738833 | 7.049015 | 7.77279 | 10.50504 | 8.235967 | 8.292553 |
| Ptom.002G.02463 | 599.738 | 289.159 | 485.972 | 200.31467 | 529.657 | 499.3333333 | 272.759 | 559.2753 | 398.062 |
| Ptom.013G.00600 | 0 | 0 | 0 | 0 | 0 | 0 | 0 | 0 | 0 |

| | | | | | | | | | |
|---|---|---|---|---|---|---|---|---|---|
| Ptom.013G.00601 | 14.8007 | 16.3342 | 33.0014 | 17.547833 | 41.0883 | 44.35683333 | 16.94966667 | 15.51443 | 20.9082 |
| Ptom.017G.00465 | 17.3951 | 28.7602 | 29.149867 | 25.430167 | 25.96575 | 29.9544 | 21.4682 | 27.5881 | 20.14037 |
| Ptom.017G.00466 | 5.17103 | 6.56598 | 6.05545 | 7.10797 | 5.88056 | 5.543103333 | 5.872366667 | 5.907663 | 5.395477 |
| Ptom.014G.00137 | 508.807 | 754.52 | 449.85433 | 624.26167 | 304 | 353.5306667 | 212.2136667 | 118.4118 | 236.1483 |
| Ptom.002G.01294 | 0.429448 | 3.63479 | 67.449233 | 3.6475967 | 35.18705 | 42.66306667 | 8.215083333 | 70.13103 | 44.46343 |
| Ptom.002G.01295 | 6.00467 | 6.27708 | 15.35421 | 6.43068 | 14.7342 | 12.3669 | 9.865456667 | 26.70287 | 12.44573 |
| Ptom.018G.01078 | 0 | 0.09527 | 8.0683767 | 0.2901107 | 0 | 0 | 0.173503333 | 0.680603 | 1.186189 |
| Ptom.014G.00353 | 0 | 0.00773 | 0.0877157 | 0.0377135 | 0.204458 | 0.298060067 | 0.590931 | 0.144023 | 0.037573 |
| Ptom.014G.00352 | 0.774756 | 2.12288 | 128.89207 | 1.6646397 | 15.57165 | 25.44396667 | 9.51266 | 0.375941 | 50.32503 |
| Ptom.018G.00629 | 0.102424 | 0.76506 | 0.426589 | 0.4113317 | 0.428254 | 0.172504333 | 0.246588667 | 0.352446 | 0.063484 |
| Ptom.006G.00835 | 1.18838 | 2.81153 | 1.433506 | 2.78452 | 0.558349 | 0.676185 | 0.599120667 | 1.603007 | 1.110152 |
| Ptom.006G.00836 | 30.0597 | 66.4952 | 20.2426 | 58.967233 | 19.03955 | 15.53883333 | 18.4913 | 22.5097 | 13.28033 |
| Ptom.005G.01312 | 4.39634 | 4.86074 | 9.26368 | 4.2848967 | 1.532499 | 2.521656667 | 9.52132 | 15.2035 | 17.58567 |
| Ptom.003G.00199 | 3.07519 | 13.3496 | 3.7601467 | 9.9344467 | 1.019249 | 1.114451667 | 21.17013333 | 2.919157 | 1.410456 |
| Ptom.018G.01072 | 0 | 1.24309 | 0 | 2.14435 | 0 | 0 | 0.435165667 | 0 | 0 |
| Ptom.018G.01071 | 0 | 0.18803 | 0.5886153 | 0.2168103 | 0 | 0.295747333 | 0.097701 | 0.151107 | 0.396969 |
| Ptom.006G.00759 | 45.1227 | 26.8466 | 12.084367 | 35.082933 | 6.86524 | 7.935706667 | 32.3618 | 19.68717 | 17.41783 |
| Ptom.006G.00758 | 0 | 0 | 0 | 0 | 0 | 0 | 0.011378933 | 0.015084 | 0 |
| Ptom.010G.01098 | 0 | 0.1813 | 0 | 0 | 0 | 0 | 0.148983167 | 0 | 0.02648 |
| Ptom.006G.01598 | 0 | 0.34596 | 0.0281081 | 9.2498033 | 0 | 0 | 62.0565 | 0.045027 | 0.053264 |
| Ptom.016G.01280 | 5.79808 | 10.9079 | 3.5804933 | 10.1729 | 2.507275 | 2.7104 | 6.559206667 | 4.265207 | 5.31091 |
| Ptom.001G.01548 | 13.7857 | 37.3998 | 0.0295427 | 36.6749 | 0.156321 | 0.117611833 | 32.02203333 | 11.66457 | 7.381057 |
| Ptom.016G.00892 | 0 | 0 | 0 | 0 | 0 | 0 | 0 | 0 | 0 |
| Ptom.002G.02263 | 27.2267 | 19.1491 | 28.9414 | 17.7146 | 26.41935 | 26.18203333 | 19.68926667 | 31.8342 | 24.4916 |
| Ptom.001G.00367 | 0 | 0 | 0 | 0 | 0 | 0 | 0 | 0 | 0 |
| Ptom.005G.01558 | 16.6448 | 18.8216 | 12.944933 | 15.613233 | 10.01239 | 7.92064 | 15.3325 | 16.38113 | 13.4669 |

| | | | | | | | | | |
|---|---|---|---|---|---|---|---|---|---|
| Ptom.005G.01557 | 0 | 0.027 | 0.0243075 | 0.0271609 | 0 | 0 | 0.021967967 | 0 | 0 |
| Ptom.011G.00792 | 15.7246 | 13.1568 | 14.694167 | 12.0041 | 23.30915 | 19.20186667 | 17.24633333 | 17.45267 | 14.63633 |
| Ptom.011G.00791 | 88.7561 | 14.3127 | 3.807706 | 28.934233 | 1.639056 | 2.64601 | 25.42513333 | 4.574575 | 6.958857 |
| Ptom.016G.01184 | 7.65832 | 21.3938 | 10.683857 | 11.0516 | 6.01966 | 6.89301 | 7.052943333 | 7.108573 | 8.38846 |
| Ptom.016G.00300 | 5.50953 | 7.93471 | 9.46867 | 8.42301 | 8.920695 | 8.16371 | 9.280923333 | 7.94488 | 7.69634 |
| Ptom.016G.00299 | 14.0522 | 15.9167 | 46.073233 | 15.694967 | 55.52195 | 40.3885 | 21.62313333 | 77.28057 | 47.25843 |
| Ptom.002G.01132 | 31.7803 | 16.7765 | 132.077 | 32.173467 | 76.17805 | 57.94196667 | 27.08226667 | 182.2663 | 142.0023 |
| Ptom.001G.02163 | 3.3205 | 1.85652 | 1.8515123 | 1.93233 | 1.337521 | 0.856903 | 1.761346667 | 1.787067 | 1.391973 |
| Ptom.001G.02164 | 0 | 0 | 0 | 0 | 0 | 0 | 0 | 0 | 0 |
| Ptom.009G.00359 | 5.96073 | 9.13015 | 4.7941233 | 9.5637333 | 6.24299 | 7.679143333 | 13.75466667 | 7.753503 | 7.5017 |

**Table S10** Gene-specific primers used in this study.

| | Primer name | Forward primer sequences ( 5' to 3' ) | Reverse primer sequences ( 5' to 3' ) |
|---|---|---|---|
| **Real-time quantitative PCR primers** | *Actin* | TTCATTTCACATCTTCCCCTTTT | GATCTCTGTGTGGGCGTCTGT |
| | *PtoNAC83* | TCCACCCTACAGACGAGGAG | GGCAAATCCCAAGGGTCAGA |
| | *PtoMYB46* | TGCATTCCCTTCTCGGCAAT | GTGATGCGTTGGATGACTGC |
| | *PtoTraB1* | CCTATGGATCCGAACCCTTCT | CTCCTTCCTCCCCATCACGA |
| | *PtoTraB2* | TAAGCTCACCACACTCGCAC | GTAGGTGGGTCGAGTGGAAA |
| | 18S rRNA | ACACGGGGAGGTAGTGACAA | CCTCCAATGGATCCTCGTTA |
| **Plasmid construction primers** | *PtoNAC83* | ATGGAGAAGCTTAATTTTGTT | TTATGGTTTTCTTCTAAAATAAGGA |
| | *PtoMYB46* | ATGGCGTCGTTTGCTCTGA | CTACCACAAGCCAATGAACTTCC |
